# School Bullying Among Left-Behind Children: The Efficacy of Art Therapy on Reducing Bullying Victimization

**DOI:** 10.3389/fpsyt.2019.00040

**Published:** 2019-02-07

**Authors:** Hu Yan, Jindong Chen, Jing Huang

**Affiliations:** ^1^School of Mental Health, Wenzhou Medical University, Wen Zhou, China; ^2^Department of Psychiatry, The Second Xiangya Hospital, Central South University, Changsha, China; ^3^Hunan Key Laboratory of Psychiatry and Mental Health, Mental Health Institute of the Second Xiangya Hospital, Chinese National Clinical Research Center for Mental Disorders (Xiangya), Chinese National Technology Institute on Mental Disorders, Central South University, Changsha, China

**Keywords:** left-behind children, school bullying, school life satisfaction, self-esteem, art therapy

## Abstract

**Background:** Left-behind children (LBC) are becoming a widespread phenomenon and face higher risks of psychological and educational problems. Our study aimed to explore school bullying in LBC and examine the effectiveness of art therapy intervention for reducing bullying victimization affecting LBC in rural areas.

**Methods:** A total of 603 children, including 272 LBC and 331 non-LBC, were sampled from 6 rural schools. Questionnaires regarding school life satisfaction, children's social anxiety, self-esteem, and school bullying were used to assess the psychological and school behavior status of these children. One Hundred and Eighty LBC who were victims of school bullying were then selected and randomly assigned to 3 groups to evaluate the effects of art therapy intervention. The interventions of art therapy and general counseling were conducted in 6 sessions over 3 months.

**Results:** Our results demonstrated LBC experienced more bullying victimization than non-LBC. Left-behind boys were more likely to be bullied than left-behind girls. LBC > 12 years old, LBC whose parents are divorced, separated, or widowed, were more vulnerable to being bullied. School bullying of LBC was affected by social anxiety scores and school life satisfaction. The bullying victimization of LBC in the art therapy group was significantly improved.

**Conclusions:** LBC suffered more school bullying than did non-LBC. Art therapy can effectively help LBC in rural primary schools to reduce their vulnerability to bullying.

## Introduction

In China, children in rural areas who experienced separation from one or both of their parents for more than 6 months are defined as left-behind children (LBC) (1). Government statistics show that more than 65 million children in rural areas have parents who have left home to search for work in the city, accounting for 37.70% of all rural children in China (2). The majority of these children are cared for by their grandparents (89.30%), mainly in the undeveloped central and western provinces (more than 90%). According to the National Bureau of Statistics of China, the number of rural-urban migrants will increase to almost 300 million people in the coming 10 years, which will substantially increase the number of LBC (3). These children are a special group, as they have a higher risk of psychological and educational problems in rural China, and their growth and development have been of general concern to society. A lacking of parental care and nurturance lead to a higher likelihood of LBC being bullied, attempting suicide, and suffering from abuse (4). A number of studies have examined the associations between parental migration and children's psychological health and social interactions, mostly suggesting an increase in the risks of psychological problems, school bullying, child abuse, and suicide (5–7).

School bullying, including physical, verbal, relational, and sexual harassment, is a worldwide phenomenon and concern (8–10). According to the recently updated national representative survey data in China, the prevalence of bullying victimization, bullying behaviors, and witnessing of bullying were 26.10, 9.03, and 28.90%, respectively, showing that school bullying has become a significant concern in China (11). Students from primary schools are more likely to be involved in school bullying compared with other pre-college school types (11). More importantly, school bullying has been associated with increased risks of displaying a number of severe and persistent problems, including low self-esteem (12), poor academic achievement, emotional issues, post-traumatic stress disorder, mental health problems (13), post-traumatic stress disorder (14), self-harm, and even suicide (15). Studies have also shown that both victims and perpetrators of school bullying can be influenced by these consequences, because students bullied in school may also be bullied in their families or communities (16).

The incidence of bullying among LBC is significantly higher than among non-left-behind children (non-LBC). LBC bullying is largely related to their living environment; the lengthy lack of care and nurturance from their parents makes them more vulnerable to being bullied by others and have bullying behavior, resulting in frequent bullying (4, 17). An increasing number of studies have shown that being bullied is associated with low life satisfaction and self-esteem level, and as LBC experienced higher levels of bullying, their school life satisfaction and self-esteem decreased (18, 19).

The high prevalence and harmful effects of school bullying in LBC mean it is critical to identify and decrease the bullying occurrences. Previous studies have found that art therapy interventions can be an effective anti-bullying tool (20). Different creative techniques have been employed in art therapy, including painting, sculpting, finger painting, and collage therapy (21, 22). Differentiated from traditional verbal psychological treatment, art therapy is not restricted by language, age, cognitive ability, disease, and so forth, which is helpful for emotional expression (23). A large number of studies have shown that art therapy plays a unique and important role in the treatment of mood disorders among teenagers, anxiety or depression of cancer patients, academic pressure, and other challenges (23–27). Moreover, many students enjoy the process of painting itself in order to express themselves. While painting, students can freely create and express their feelings and thoughts artistically. It has been shown to help students better understand bullying through the process of drawing on their individual experiences (28). To date, little research has been conducted on art therapy as an intervention for LBC bullying behavior; more studies could be performed in the future to allow for a better understanding and control of school bullying.

The purpose of this study was to explore the life satisfaction and bullying victimization among LBC from 6 rural schools in Hunan, Henan, Liaoning, and Guangxi provinces of China. We also want to assess the effectiveness of art therapy intervention for reducing the bullying victimization affecting these children who have been exposed to internal migration.

## Methods

### Inclusion Criteria

A cross-sectional study was performed in six primary schools in Hunan, Henan, Liaoning, and Guangxi provinces from June 2017 to April 2018. The provinces and schools were selected because of their relatively behindhand economic and natural limit, huge population along with rural-urban migration. Fifth grade children were randomly sampled from three classes in each school. All students in each selected class were included in the survey. A total of 603 children from 18 fifth grade classes were included in the study, including 272 LBC (45.11%) and 331 non-LBC (54.89%). The average age was 10.92 ± 0.79. Among the 272 LBC were 155 girls and 117 boys.

In this study, a questionnaire survey was administered to 603 children that included general information (individual characteristics, family, and school information), children's mental status, and their experiences of bullying victimization. Questionnaires regarding school life satisfaction, children's social anxiety, self-esteem, and school bullying were used to assess the psychological and school life status of the included children.

We then assessed the effects of art therapy intervention among LBC with regard to school bullying. The children with the top 30 bullying scores from LBC from each of the schools were included in the therapy, and they were randomly divided into three groups. A total of 180 LBC from 6 schools were selected for intervention assessments. The subjects were randomized into an art therapy group, a general counseling group, and a control group. Of these, 60 LBC were included in an art therapy group, 60 LBC were included in a general counseling group, and 60 LBC were included in a control group without any psychological interventions. The general information from the 3 groups was compared with a chi-square test.

Four children in the art therapy group, 5 children in the general counseling group, and 2 children in the control group withdrew from the study before its completion. Thus, 169 LBC were included in the final intervention evaluations (29 boys and 27 girls in the art therapy group, mean age 11.25 ± 0.67; 25 boys and 30 girls in the general counseling group, mean age 10.85 ± 0.76; 34 boys and 24 girls in the control group, mean age 11.09 ± 0.73).

The study was approved by research ethics committee of Wenzhou Medical University and supported by the Hunan provincial social science foundation (16YBA363). All participants provided oral assent before the survey. Written informed consent were obtained from school administrators and children's parents/guardians. The study was conducted in accordance with the Declaration of Helsinki.

### Measures

The modified Brief Multidimensional Students' Life Satisfaction Scale-Peabody Treatment Progress Battery (BMSLSS-PTPB: Youth) was used to assess the children's school life satisfaction (29, 30). The scale is composed of 13 items scored on a scale from 1 (strongly disagree) to 4 (strongly agree), and a total score is calculated by summing the 13 items. The higher the score, the higher school life satisfaction. In this study, the scale of the Cronbach's alpha coefficient is 0.832, and the KMO (Kaiser-Meyer-Olkin) coefficient is 0.847.

From the Social Anxiety Scale for Children-Revised (SASC-R) (31, 32), which was developed by La Greca to screen children's social anxiety symptoms, we selected 10 items to assess social anxiety in these children. Each item is rated on a 3-point Likert scale, ranging from 1 (not at all) to 3 (all the time). Factor analysis of the scale yielded two separate factors, including the following: fear of negative evaluations (6 items), and social avoidance and distress (4 items). The total score ranged from 10 to 30, with higher scores indicating more severe social anxiety. The internal consistency of the scale was acceptable; the Cronbach's alpha coefficient is 0.860, and the KMO coefficient is 0.904.

The children's self-esteem level was measured by the Rosenberg self-esteem scale (RSES), which was developed by Rosenberg in 1965 to assess the overall feeling of self-worth and self-acceptance in teenagers (33, 34). Ten items make up the scale, with 5 positively worded and 5 negatively worded statements. Each item is answered on a four-point scale—from strongly agree (1 point) to strongly disagree (4 points). The total score range is 10–40 points in which higher scores reflect higher levels of self-esteem. The Cronbach's alpha coefficient in this sample was 0.773, the KMO coefficient was 0.811.

The children's bullying experience was measured by the Revised Olweus Bully/Victim Questionnaire (4, 8, 35, 36). Of the 40 items, we selected 24 to assess these children's bullying victimization and bullying behaviors. The questionnaire was divided into two main dimensions: being bullied and bullying others, each with 12 items. Each item is rated on a 5-point scale, ranging from 1 (never) to 5 (several times a week). In this study, the scale of the Cronbach's alpha coefficient is 0.947, KMO coefficient of 0.917.

### Method for Measuring

A teacher with a psychological background was selected in each school. Six teachers in total were trained by the researchers in advance to collect data and develop group guidance, as well as to assist the students to finish the questionnaire survey in the school computer labs according to the instructions. The computer-assisted questionnaires could only be submitted with all essential information completed.

The interventions of art therapy and general counseling were conducted in 6 sessions over 3 months. Interventions were scheduled every 2 weeks. The art therapy group mainly used different theme painting activities to help students to understand what bullying is and how to cope with it. Tasks included a self-portrait, drawing of our group and our story, drawing people and things that make me feel warm, drawing my strengths, drawing the future dreams, and drawing my friends. In the general counseling group, children need to recall their experiences of being bullied, and talk about what to do when being laughed by others. This activity relied more heavily on language expression of the children. No intervention was performed in the control group. All the LBC included in the three groups completed questionnaires regarding school life satisfaction, children's social anxiety, self-esteem, and school bullying at the initiation and end of the intervention study. The activities of the intervention groups are shown in Supplement Table 1.

### Statistical Analysis

SPSS22.0 was used for statistical analysis. Chi-square tests were performed to evaluate differences in categorical variables among LBC and non-LBC. We investigated the association between school social satisfaction, social anxiety, self-esteem, and bullying victimization using logistic regression analysis. The statistical significance was considered at *p* < 0.05 for two-sided tests.

## Results

### Comparison of School Life Satisfaction, Self-Esteem, and School Bullying Between LBC and Non-LBC

First, we compared the school life satisfaction, self-esteem, and school bullying status between LBC and non-LBC. Table 1 shows the total score of school life satisfaction, self-esteem, and bullying (including bullying victimization and bullying behavior) among LBC and non-LBC. LBC showed higher school life satisfaction (*p* < 0.01) and lower levels of self-esteem (*p* < 0.05); LBC also suffered more bullying victimization than did non-LBC (*p* < 0.01), and there was no significant difference on scores for bullying others (*p* > 0.05). Table 2 shows that life satisfaction among the left-behind girls was significantly higher than that of left-behind boys (*p* < 0.05). Left-behind boys suffered more school bullying (*p* < 0.001) and had more bullying behavior to other children (*p* < 0.05).

**Table 1:** The comparison of school life satisfaction, social anxiety, self-esteem and school bullying differences between LBC and non-LBC (*n* = 603).

	**School life satisfaction**	**Fear of negative evaluation**	**Social avoidance and Distress**	**Social anxiety**	**Self-esteem**	**Bullying victimization**	**Bullying behavior**
LBC (*n* = 272)	3.084 ± 0.43	1.613 ± 0.45	1.537 ± 0.44	3.150 ± 0.80	27.749 ± 5.23	17.746 ± 7.24	14.289 ± 5.06
non-LBC(*n* = 331)	2.982 ± 0.51	1.516 ± 0.42	1.500 ± 0.44	3.016 ± 0.76	28.722 ± 4.06	16.109 ± 6.46	14.018 ± 5.64
*T*	2.647^**^	2.738^**^	1.023	2.115^*^	−2.557^*^	2.860^**^	0.612

* *p < 0.05*,

** *p < 0.01. LBC, Left-behind Children*.

**Table 2:** The comparative analysis of school life satisfaction, social anxiety, self-esteem and school bullying in LBC.

	**School life satisfaction**	**Fear of negative evaluation**	**Social avoidance and distress**	**Social anxiety**	**Self-esteem**	**Bullying victimization**	**Bullying behavior**
Boys (*n* = 117)	3.011 ± 0.41	1.620 ± 0.50	1.588 ± 0.47	3.209 ± 0.86	28.388 ± 4.02	19.617 ± 8.30	15.161 ± 5.937
Girls (*n* = 155)	3.141 ± 0.44	1.612 ± 0.42	1.500 ± 0.41	3.112 ± 0.74	29.027 ± 4.08	16.271 ± 5.91	13.611 ± 4.14
*t*	−2.483^*^	0.155	1.608	0.979	−1.270	3.649^***^	2.457^*^

* *p < 0.05*,

*** *p < 0.001*.

### LBC's School Life Satisfaction, Self-Esteem, and School Bullying Situations

Compared to LBC under the age of 12, LBC older than 12 suffered more bullying (*p* < 0.01). Although with increased chance of being bullied by others, the total score of bullying behavior in LBC older than 12 showed no significant difference compared with LBC under the age of 12 (*p* > 0.05). Parents' marital status had an important influence on LBC's bullying victimization and bullying behavior. LBC whose parents were divorced, separated, or widowed suffered more bullying than LBC whose parents were married (*p* < 0.01). Parents' marital status also greatly impacted children's bullying behavior; LBC whose parents were married engaged in less bullying behavior than other LBC (*p* < 0.01) (Supplement Table 2).

Further analysis revealed that school life satisfaction (*p* < 0.01), self-esteem (*p* < 0.01), and bullying victimization among LBC were significantly negative correlated; the bullying behavior of LBC was also significantly negative correlated with school life satisfaction (*p* < 0.01) and self-esteem (*p* < 0.01) (Supplement Table 3).We performed a step-wise regression analysis with school life satisfaction and social anxiety as independent variables and the total score of being bullied as the dependent variable (Supplement Table 4).

The primary data for school life satisfaction, self-esteem, and bullying victimization have been preprocessed to get zero-centered data for further analysis. Then we used linear regression analysis for the mediating effect analysis. First, variables such as gender, age, and marital status of parents, which significantly influenced the total score of being bullied, were controlled; the total score of bullying victimization was regarded as the dependent variable and school life satisfaction as the prediction variable. It was found that school life satisfaction can be an effective expectant variable for being bullied (β = −4.990, *p* < 0.001). We then used self-esteem as the dependent variable and school life satisfaction as the prediction variable; it was found that school life satisfaction had an effect on the prediction of self-esteem (β = 3.582, *p* < 0.001). Finally, we used the score of bullying victimization as the dependent variable, and school life satisfaction and self-esteem were simultaneously put into the regression analysis; the results show that when the self-esteem variable was included, the prediction of school life satisfaction for being bullied dropped, but was still significant (β = −4.145, *p* < 0.001). Self-esteem also predicted the total score of being bullied (β = −0.250, *p* < 0.05), indicating that self-esteem acted as a partial mediator between school life satisfaction and the total score of being bullied.

We further conducted mediating effect analysis to evaluate the influence of school life satisfaction and self-esteem on bullying behavior. After controlling for statistically significant variables such as gender, age, and the marital status of parents, the total score of bullying behavior was regarded as the dependent variable and school life satisfaction as the prediction variables; it was found that school life satisfaction can predict bullying behavior (β = −2.567, *p* < 0.001). We used the score of bullying behavior as the dependent variable, and school life satisfaction and self-esteem were simultaneously included in the regression analysis; the results show that when self-esteem was added, the prediction of school life satisfaction for bullying behavior dropped, but was still significant (β = −2.106, *p* < 0.05). Self-esteem predicted the total score of bullying behavior without significance (β = −0.131, *p* > 0.05), indicating that self-esteem did not partially mediate between school life satisfaction and the total score of bullying behavior (Table 3).

**Table 3:** Mediating effect analysis of self-esteem on school life satisfaction, bullying victimization and bullying behavior in LBC (*n* = 258).

**Process**	**Independent variable**	**Dependent variable**	**β**	***T***	***R^**2**^***	**Δ*R^**2**^***	***F***
1	School life satisfaction	Bullying victimization	−4.990	−4.957	0.088	0.084	24.575^***^
2	School life satisfaction	Self-esteem	3.582	6.756	0.151	0.148	45.640^***^
3	School life satisfaction	Bullying victimization	−4.145	−3.797	0.106	0.099	14.830^***^
	Self-esteem	–	−0.250	−2.116	–	–	–
1	School life satisfaction	Bullying behavior	−2.567	−3.567	0.048	0.044	12.723^***^
2	School life satisfaction	Self-esteem	3.582	6.756	0.151	0.148	45.640^***^
3	School life satisfaction	Bullying behavior	−2.106	−2.609	0.058	0.050	7.535^***^
	Self-esteem		−0.131	−1.501	–	–	–

*** *p < 0.001*.

### Effects of Art Therapy on Reducing School Bullying Victimization of LBC

A total of 180 LBC from 6 schools were selected for intervention assessments. no statistically significant difference in nationality, age, gender, or only child status was found among the 3 groups of LBC (*p* > 0.05, Supplemental Table 5).

As Table 4 suggests, there was no statistically significant difference on the total score of bullying victimization, school life satisfaction, and self-esteem among the 3 groups before the intervention (*p* > 0.05). There was no significant improvement in bullying victimization in the general counseling intervention group and control group during the intervention period (*p* > 0.05). After the art therapy intervention, the LBC in the art therapy group showed significantly reduced bullying victimization (*p* < 0.05) and significantly increased self-esteem level (*p* < 0.05).

**Table 4:** Pre-test and post-test of bullying victimization in art therapy group, general counseling group, and control group (*n* = 169).

**Group**	**Time**	**School life satisfaction**	**Fear of negative evaluation**	**Social avoidance and distress**	**Social anxiety**	**Self-esteem**	**Bullying victimization**
Art therapy (*n* = 56) (a)	Pretest	2.917 ± 0.44	1.736 ± 0.50	1.696 ± 0.47	3.433 ± 0.90	28.429 ± 4.06	23.018 ± 9.44
	Posttest	3.052 ± 0.50	1.678 ± 0.51	1.571 ± 0.49	3.250 ± 0.92	30.071 ± 4.91	19.482 ± 9.64
*t*		−1.835	0.668	1.466	1.157	−2.402^*^	2.085^*^
General counseling (*n* = 55) (b)	Pretest	3.056 ± 0.44	1.605 ± 0.43	1.500 ± 0.42	3.105 ± 0.79	29.291 ± 4.86	20.891 ± 8.95
	Posttest	3.134 ± 0.54	1.615 ± 0.52	1.509 ± 0.51	3.124 ± 0.98	30.346 ± 4.83	18.746 ± 10.01
*t*	–	−1.071	−0.156	−0.114	−0.148	−1.643	1.938
control (*n* = 58) (c)	Pretest	3.018 ± 0.41	1.613 ± 0.49	1.571 ± 0.47	3.187 ± 0.90	27.421 ± 3.73	21.193 ± 7.42
	Posttest	3.024 ± 0.43	1.497 ± 0.48	1.491 ± 0.46	2.989 ± 0.83	28.912 ± 4.33	20.155 ± 7.81
*t*	–	−0.125	−0.121	0.374	0.109	−1.979	1.104
*F1*	–	1.551	3.257^*^	3.084^*^	3.348^*^	2.731	0.991
Pairwise comparisons	–	–	a/b; a/c	a/b; a/c	a/b; a/c	–	–
*F2*	–	0.755	1.915	0.422	1.177	1.480	0.333

* *p < 0.05. F1 refers to comparison of Baseline scores of 3 groups. F2 refers to comparison of different intervention effect*.

## Discussion

Our study found that LBC were more likely to be bullying victims than were non-LBC. It is plausible that because they do not have parents in their daily lives, they lack parental care and companionship, security, or self-confidence, and are thus more likely to become subject to bullying. Previous research found that LBC had more emotional, social, and behavioral problems than non-LBC (37). This study found that the social anxiety of LBC was significantly higher than that of non-LBC, which was consistent with Zhao's results (7). LBC were more fearful of negative evaluation and the score of self-esteem among them was significantly lower than that of non-LBC.

Compared to left-behind girls, left-behind boys experienced more bullying victimization, but also engaged in more bully behaviors. Left-behind girls experienced a higher school life satisfaction. According to Liao's results, left-behind girls had better overall academic scores and were more likely to obey school rules, which made them more popular among teachers and classmates and less likely to be bullied, namely that left-behind girls had more coping mechanisms for school bullying than did boys (38). Besides, boys score considerably higher than girls in explicit aggression in developmental psychology (39); LBC who were 12 years old and above suffered more bullying; this may because the average age of fifth grade students is 11, so fifth grade LBC who are 12 years old are developmentally delayed and more susceptible to be bullied by others. LBC with divorced, widowed, or separated parents suffered more school bullying victimization and engaged in more bullying behaviors than LBC whose parents were married. Numerous studies have shown that the integrity of family is quite important to children's mental health, and that the children of divorced parents suffer mental trauma and experience more emotional and behavioral problems than their peers (40–42). Whether LBC were only children or had siblings was not significantly correlated with school bullying behavior; this may be because only children also have the support of friends or other relatives.

We found that school life satisfaction and social anxiety scores had important effects on LBC's bullying victimization. The possible reason may be that school life is quite important for primary and secondary school students; students with higher school life satisfaction get along better with peers, which makes them less likely to be bullied. Studies have found LBC with higher social anxiety experience stronger loneliness, which makes them less likely to get help from others when being bullied (20). This study also found that self-esteem played a partial intermediary role between social anxiety and school bullying victimization, as well as between school life satisfaction and school bullying victimization. Improving the level of self-esteem may help reduce LBC's social anxiety and chances of being bullied (12).

Three intervention groups were evaluated for reducing bullying victimization. In the general counseling group, members completed the activities through telling their own story and expressing themselves. After six sessions, their social anxiety had been reduced, to a certain extent, but their bullying situations had not improved. Possible reasons for this may be that the intervention of the general counseling group mainly relied on the students speaking up and vocalizing their thoughts, which is challenging and embarrassing for some students. Some children may not have wanted to express their internal feelings in front of others, and it could be hard for them to clearly describe their emotions and feelings. In addition, some children feel stressed as they recall school bullying that happened to them, and cannot think reasonably if they do not have a relaxed attitude. In our study, 5 children discontinued the intervention in the general counseling group because they were uncomfortable speaking about their feelings.

Compared to the LBC in the other two groups, the LBC in the art therapy group experienced an improved social life and significantly reduced bullying victimization. For primary school students in the fifth grade, incomplete language skills may be a barrier for them to participate in the general counseling group, which requires much self-expression; thus, art therapy becomes an ideal and alternative psychological guidance intervention. As one of the earliest forms of psychotherapy, art therapy is a creative method of expression and communication that has been employed in many clinical and other settings with diverse populations (24). Different interventions and sessions have been designed through drawing and painting to facilitate communication between art therapists and subjects, in order to achieve therapeutic objectives. Researchers have found that the integration of art therapy in classrooms made students more open to instruction and change, which helped them to benefit more from the therapeutic approach. Art therapy can produce meaningful art projects, empower students, improve interpersonal relationships, and help students achieve greater self-esteem. It has also shown advantages for students in coping with social issues, especially school bullying (43).

Several limitations remain in our study. First, this study only investigated the bullying phenomenon (including bullying victimization and bullying behavior) of LBC in fifth grade students, so the age range is relatively small. Second, our study explored the relationships between bullying and variables such as demographic characteristics, school life satisfaction, social anxiety, and self-esteem, but LBC's coping mechanisms for bullying situation were not explored. Future studies can examine a larger age range to investigate bullying victimization and assess the coping mechanisms LBC use, this can help to better address school bullying victimization problems among LBC. The findings can be more reliable and accurate if we used longitudinal study method and different evaluation methods besides self-report questionnaires. In addition, this study investigated the effects of art therapy and general counseling interventions to improve the bullying victimization of LBC; the effects of a wider variety of interventions to reduce bullying behavior in LBC should be explored in future studies. Finally, only 6 sessions of interventions (art therapy group and general counseling group) were conducted, and the predictive value of our results for long-term effects remains to be further validated. There is a need to explore the most effective psychological intervention models, so long-term and sustainable psychological interventions should be investigated in further studies.

To conclude, our results indicate that LBC suffer more bullying and engage in more bullying behavior than do non-LBC. Left-behind boys, LBC above 12 years old, and LBC whose parents are divorced, separated, or widowed were more vulnerable to being bullied. LBC's bullying behavior was correlated with social anxiety scores and school life satisfaction. In addition, self-esteem played a partial intermediary role between social anxiety and bullying behavior, as well as between life satisfaction and bullying behavior.

## Author Contributions

HY and JC reviewed literature, designed the study, prepared the manuscript, performed data extraction and quality assessment. JH supervised all the work.

### Conflict of Interest Statement

The authors declare that the research was conducted in the absence of any commercial or financial relationships that could be construed as a potential conflict of interest.

## Abbreviations

LBC: Left-behind Children
BMSLSS-PTPB: Youth: Brief Multidimensional Students' Life Satisfaction Scale-Peabody Treatment Progress Battery
KMO: Kaiser-Meyer-Olkin
RSES: Rosenberg self-esteem scale
SASC-R: Social Anxiety Scale for Children-Revised.
