# Supplementary material for: School Bullying Among Left-Behind Children: The Efficacy of Art Therapy on Reducing Bullying Victimization
Source: Front Psychiatry. 2019 Feb 7;10:40. doi: 10.3389/fpsyt.2019.00040 (PMC6374290; doi:10.3389/fpsyt.2019.00040)
Supplement: Supplementary file 1 [file Table_1.DOCX]

Supplement Table S1. Intervention methods of three groups

| Sequence | Theme | Aim | Plan for art therapy group | Plan for general counseling group |
| --- | --- | --- | --- | --- |
| First | Gathering together | 1. Group leaders and members get to know each other, promote their familiarity, and establish a good relationship of group psychological counseling.  2. Members of the group know each other.  3. Make the group Agreement | 1. Start: introduce yourself and introduce the theme of group activities  2. Warm up: the wind blows  3. My self-portrait (self-introduction)  4. Draw our group  5. Affirm the oath  6. Summarize and share | 1. Start: introduce yourself and introduce the theme of group activities  2. Warm up: the wind blows  3. True or false (self-introduction)  4. Name the group  5. Affirm the oath  6. Summarize and share |
| Second | What is School bullying？ | 1. Help left-behind children understand what School bullying is  2. Know whether your behavior belongs to School bullying  3. Know whether you are being aggrieved by School bullying | 1. Start: introduce the theme of the activity, and repeat the group rules  2. Warm up: throwing dolls (small balls)  3. What is campus violence? "-- dam keeper  4. Draw our story  5. Share and summarize | 1. Start: introduce the theme of the activity, and repeat the group rules  2. Warm up: throwing dolls (small balls)  3. What is campus violence? "-- dam keeper  4. Tell our story  5. Share and summarize |
| Third | Face bullying with courage | 1. Attach importance to school bullying actively and courageously resist it;  2. Asking for help | 1. Start：introduce the theme of group activities and repeating the group rules  2. Warm up: have a massage to relax  3. Role playing: rebel and shout loudly  4. Draw people and things that make me feel warm  5. Share and summarize | 1. Start：introduce the theme of group activities and repeating the group rules  2. Warm up: have a massage to relax  3. Role playing: rebel and shout loudly  4. Asking for help from teachers, parents and friends  5. Share and summarize |
| Fourth | Everybody can do something | 1. Help students cope with ridicule.  2. Learn to find and develop your own strengths and interests by watching video. | 1. Start：introduce the theme of the activity  2. Warm up: sit up  3. how to cope with ridicule  4. I was born with talents. Draw my strengths  5. Share and summarize | 1. Start：introduce the theme of the activity  2. Warm up: sit up  3. how to cope with ridicule  4. A piece of chalk  5. Share and summarize |
| Fifth | Let your dreams fly | 1. Help members to understand their own shortcomings and how to improve them.  2. Think about the future and inspire action with your dreams. | 1. Start: introduce the theme of group activities  2. Warm up: poor cat  3. Paint your future dreams  4. Draw the lifeline  5. Summarize and share | 1. Start: introduce the theme of group activities  2. Warm up: peach blossoms bloom  3. Talk about your future dreams  4. Share the life plan  5. Summarize and share |
| Sixth | Goodbye, school bullying | Strengthen the relationship between the members of the group, get rid of the influence of school bullying, keep away from School bullying and reduce the occurrence of school bullying. | 1. Start by introducing the theme of group activities  2.Warm up: peach blossoms bloom  3. Draw my self-portrait and my friends  4. Goodbye, the bad things  5. Thanks for having you  6. Summarize and share | 1. Start by introducing the theme of group activities  2.Warm up: peach blossoms bloom  3. My change  4. Goodbye, the bad things  5. Thanks for having you  6. Summarize and share |

Supplement Table S2. The comparison of different age group, parental marital status and school bullying in LBC

| classification | Groups | Bullying victimization | Bullying behavior | Post Hoc Test |
| --- | --- | --- | --- | --- |
| Age (n=260) | ≤10 (n=95, a) | 17.737±6.69 | 14.660±5.64 | a/c; b/c |
|  | 11 (n=116, b)  ≥12 (n=49, c) | 16.526±5.95  20.653±9.90 | 13.655±3.72  15.041±6.34 | - |
| F  parental marital status (n=258)  F | Married (n=199, a)  Divorced (n=25, b)  Other (n=34, c) | 5.809**  17.015±6.42  20.000±9.28  20.539±9.27  4.851** | 1.693  13.835±4.21  14.360±5.30  16.800±7.96  5.236** | a/c; b/c |

Supplement Table S3. Associations between School life satisfaction, Social anxiety and Self-esteem with School bullying in LBC

| Coefficient of production-moment correlation | School life satisfaction | Self-esteem | Bullying victimization |
| --- | --- | --- | --- |
| Self-esteem | .388^**^ |  |  |
| Bullying victimization | -.296^**^ | -.230^**^ |  |
| Bullying behavior | -.219^**^ | -.177^**^ | .576^**^ |

Supplement Table S4. Regression Analysis Table of School life satisfaction, Social anxiety and School bullying

|  | Independent Variable | Dependent Variable | | *β* | *t* | *R^2^* | *ΔR^2^* | *F* |
| --- | --- | --- | --- | --- | --- | --- | --- | --- |
| Input | Social anxiety | Bullying behavior | 2.727 | | 4.895 | 0.170 | 0.164 | 25.542*** |
|  | School life satisfaction |  | -3.405 | | -3.322 |  |  |  |
| Input | School life satisfaction | Bullying victimization | -2.032 | | -2.643 | 0.070 | 0.062 | 9.227*** |
|  | Social anxiety |  | 0.971 | | 2.339 |  |  |  |

Supplement Table S5. Demographic characteristics of art therapy group, general counseling group, and control group

| Group | Number | Nationality | | Age | | | Gender | | Only child | |
| --- | --- | --- | --- | --- | --- | --- | --- | --- | --- | --- |
|  |  | Han | Minority | 10 | 11 | 12 | Male | Female | Yes | No |
| art therapy | 56 | 45 | 11 | 7 | 28 | 21 | 29 | 27 | 15 | 41 |
| general counseling | 55 | 45 | 10 | 20 | 23 | 12 | 25 | 30 | 15 | 40 |
| control | 58 | 43 | 15 | 13 | 27 | 18 | 34 | 24 | 8 | 50 |
| *χ*^2^ |  | 1.131 | | 9.423 | | | 1.964 | | 3.832 | |
| *P* |  | 0.568 | | 0.051 | | | 0.375 | | 0.147 | |
